# Resistance Mutations to BTK Inhibitors Originate From the NF-κB but Not From the PI3K-RAS-MAPK Arm of the B Cell Receptor Signaling Pathway

**DOI:** 10.3389/fimmu.2021.689472

**Published:** 2021-06-10

**Authors:** C. I. Edvard Smith, Jan A. Burger

**Affiliations:** ^1^Department of Laboratory Medicine, Karolinska Institutet (KI), Huddinge, Sweden; ^2^Department of Leukemia, University of Texas MD Anderson Cancer Center, Houston, TX, United States

**Keywords:** Btk, ibrutinib, B cell receptor, chronic lymphocytic leukemia, mantle cell lymphoma, Waldenström’s macroglobulinemia, resistance mutation, CARD11

## Abstract

Since the first clinical report in 2013, inhibitors of the intracellular kinase BTK (BTKi) have profoundly altered the treatment paradigm of B cell malignancies, replacing chemotherapy with targeted agents in patients with chronic lymphocytic leukemia (CLL), mantle cell lymphoma (MCL), and Waldenström’s macroglobulinemia. There are over 20 BTKi, both irreversible and reversible, in clinical development. While loss-of-function (LoF) mutations in the *BTK* gene cause the immunodeficiency X-linked agammaglobulinemia, neither inherited, nor somatic *BTK* driver mutations are known. Instead, BTKi-sensitive malignancies are addicted to BTK. BTK is activated by upstream surface receptors, especially the B cell receptor (BCR) but also by chemokine receptors, and adhesion molecules regulating B cell homing. Consequently, BTKi therapy abrogates BCR-driven proliferation and the tissue homing capacity of the malignant cells, which are being redistributed into peripheral blood. BTKi resistance can develop over time, especially in MCL and high-risk CLL patients. Frequently, resistance mutations affect the BTKi binding-site, cysteine 481, thereby reducing drug binding. Less common are gain-of-function (GoF) mutations in downstream signaling components, including phospholipase Cγ2 (PLCγ2). In a subset of patients, mechanisms outside of the BCR pathway, related e.g. to resistance to apoptosis were described. BCR signaling depends on many proteins including SYK, BTK, PI3K; still based on the resistance pattern, BTKi therapy only selects GoF alterations in the NF-κB arm, whereas an inhibitor of the p110δ subunit of PI3K instead selects resistance mutations in the RAS-MAP kinase pathway. BTK and PLCγ2 resistance mutations highlight BTK’s non-redundant role in BCR-mediated NF-κB activation. Of note, mutations affecting BTK tend to generate clone sizes larger than alterations in PLCγ2. This infers that BTK signaling may go beyond the PLCγ2-regulated NF-κB and NFAT arms. Collectively, when comparing the primary and acquired mutation spectrum in BTKi-sensitive malignancies with the phenotype of the corresponding germline alterations, we find that certain observations do not readily fit with the existing models of BCR signaling.

## Introduction

The sequence of the *BTK* gene was reported in 1993 by two independent research teams, as a result of a hunt for the genetic cause of X-linked agammaglobulinemia, XLA (1, 2), and a search for novel tyrosine kinases (3). Given that patients with XLA are essentially devoid of mature B lymphocytes it was apparent already prior to the gene cloning that if it were possible to inhibit the product of the gene mutated in XLA, such drugs could become highly useful for targeting B cells.

However, it was not until 2007 that a compound was selected for clinical development, the first-in-class BTKi ibrutinib (4–6). Patients with diverse B cell malignancies were enrolled in the initial Phase 1 ibrutinib trial, which reported about particularly high response rates in patients with CLL and MCL (7), and from then on, the development of BTKi has been no less than astounding.

Focusing on CLL, the first ibrutinib trial demonstrated high response rates (8), and very durable effects in treatment naïve patients (7-year progression-free survival 83%), while relapsed/refractory patients had shorter remissions (7-year progression-free survival 34%) (9). Subsequently, a series of randomized clinical trials demonstrated major improvement in progression-free survival (PFS) in CLL patients (untreated or previously treated) receiving ibrutinib (alone or with CD20 antibodies), as compared with previous standard therapies, most of them chemotherapy-based (10–13). Based on these data, ibrutinib and other BTK inhibitors have become the preferred CLL treatment to date. A similar development has taken place in Waldenström’s macroglobulinemia, WM (14).

At least 22 BTKi are in clinical development (15) and sales have already placed BTKi among the most commonly sold cancer drugs worldwide (16).

While the first approved drugs, ibrutinib, acalabrutinib and zanubrutinib, bind irreversibly to a cysteine in the catalytic domain, there are now several compounds, which tether reversibly. Many of these are investigated as treatment of autoimmune and inflammatory disorders (15). They also are explored as a treatment for patients with resistance mutations to irreversible inhibitors (17, 18). The concept of irreversible kinase inhibitors was relatively novel around the time when BTKi were first generated (19). A covalent bond is formed between the BTKi, at the electrophilic site, and its target where the cysteine 481 serves as the corresponding nucleophile. Since only few kinases carry a corresponding cysteine residue this likely limits off-target side effects (15, 20–22). A more detailed description of the interaction between various BTKi and the catalytic domain of BTK is found in an accompanying paper (23).

## B Cell Malignancies Responding to BTKi Depend on BCR Signaling

To date, the responsiveness to BTKi has been studied in all major B cell malignancies and the emerging pattern is that sensitive malignancies are dependent on BCR signaling for their growth and survival. For example, in CLL, the importance of the BCR pathway was already emphasized prior to the clinical use of BTKi (24), whereas in mantle cell lymphoma (MCL) and Waldenström’s macroglobulinemia (WM) the dependence of the malignant B cells on BCR and/or BTK was mostly discovered later (25, 26).

In CLL, it was reported as early as in 1972 (27) that the specificity of the monoclonal surface immunoglobulins (IG) was not random. In the 1990s, it was noted that CLL cells express a restricted repertoire of IG heavy variable (IGHV) genes (28–30). During that time, it became obvious that the IG gene repertoire of CLL is non-random and, moreover, that a major fraction of CLL patients carried IGHV somatic hypermutations (SHM) (31). Interestingly, the SHM status of the IGHV genes is a reliable predictor for patient survival, as reported in two seminal publications by Stevenson, Chiorazzi, and colleagues (32, 33). Patients with mutated IGHV genes (‘IG-mutated’ CLL, M-CLL; 50-60% of cases) generally have more indolent disease, whereas those with unmutated IGHV genes (‘IG-unmutated’ CLL, U-CLL; 30-40% of cases) typically have more rapidly progressive disease and respond poorly to chemotherapy-based treatment (32, 33). Importantly, BTKi therapy overcomes the negative prognostic impact of U-CLL, based on longer follow-up of M-CLL and U-CLL patients treated with ibrutinib (34). Moreover, stereotyped BCR, defined as the existence of (quasi)identical BCR in different CLL patients has been another discovery highlighting the importance of the BCR and common antigens in CLL pathogenesis (35). Over the last years it was established that a large fraction of CLL, at least 41%, expressed stereotyped BCR, which is remarkable considering the extremely low probability of randomly finding identical VH CDR3 sequences in different people (36, 37). Also in MCL there is a bias in the VH usage (38) demonstrating the involvement of the BCR in these lymphomas.

As described in the section *“Recurrent Primary Mutations in B Cell Malignancies Responding to BTKi”*, BCR-mediated signaling differs in certain other BTKi-sensitive malignancies, such as WM. Here, the formation of a constitutively active intracellular signaling hub is the driving force rather than stereotypic IG at the membrane.

## Not All B-Cell Malignancies Respond to BTKi

In the *activated B-cell-like* (ABC) subtype of diffuse large B-cell lymphoma (DLBCL) an RNA interference genetic screen revealed that BTK is essential for the survival of lymphoma cells (39) with wild-type *Caspase recruitment domain family, member 11* (CARD11). Thus, while DLBCL tumors belonging to the group of *germinal center B cell-like* (GCB) are unresponsive to BTKi, the ABC subtype can be sensitive (40), although only subsets respond, and responses generally are short. Similarly, only few patients with follicular lymphoma responded transiently (7). Furthermore, when CLL is affected by Richter’s transformation additional genetic changes cause the tumor to become unresponsive to BTKi (41, 42). Thus, unless the tumor is dependent on BCR signaling sensitivity to BTKi is not expected.

## The BCR Signaling Pathway and the Critical Role of NF-κB

In classical experiments in which the BCR was genetically disabled in adult mice it was demonstrated that B-cells lacking this receptor could not survive (43), and this contributed to the idea of ‘tonic signaling’. Later this concept was also recapitulated for distinct *signaling* components of the BCR (44). Particularly in B cells, NF-κB serves as a survival factor and tonic signaling achieves sufficient NF-κB activity to prevent apoptotic events from occurring (45). This was recently corroborated at the single-cell level, by combining transcriptome profiling and chromatin mapping in BTKi-treated CLL patients. A sharp decrease of NF-κB binding was found, followed by the acquisition of a quiescence-like gene signature (46). Subsequently there was reduced chromatin accessibility at binding sites of the transcription factors regulated by NF-κB, such as PU.1. This is of interest, since mutation of the PU.1 binding site in the *BTK* promoter causes XLA (47–49), and, furthermore, because BTK uses a positive autoregulatory feedback mechanism to stimulate transcription from its own promoter *via* NF-κB (50).

## Resistance Mutations Predominantly Affect BTK

**Figure 1:**
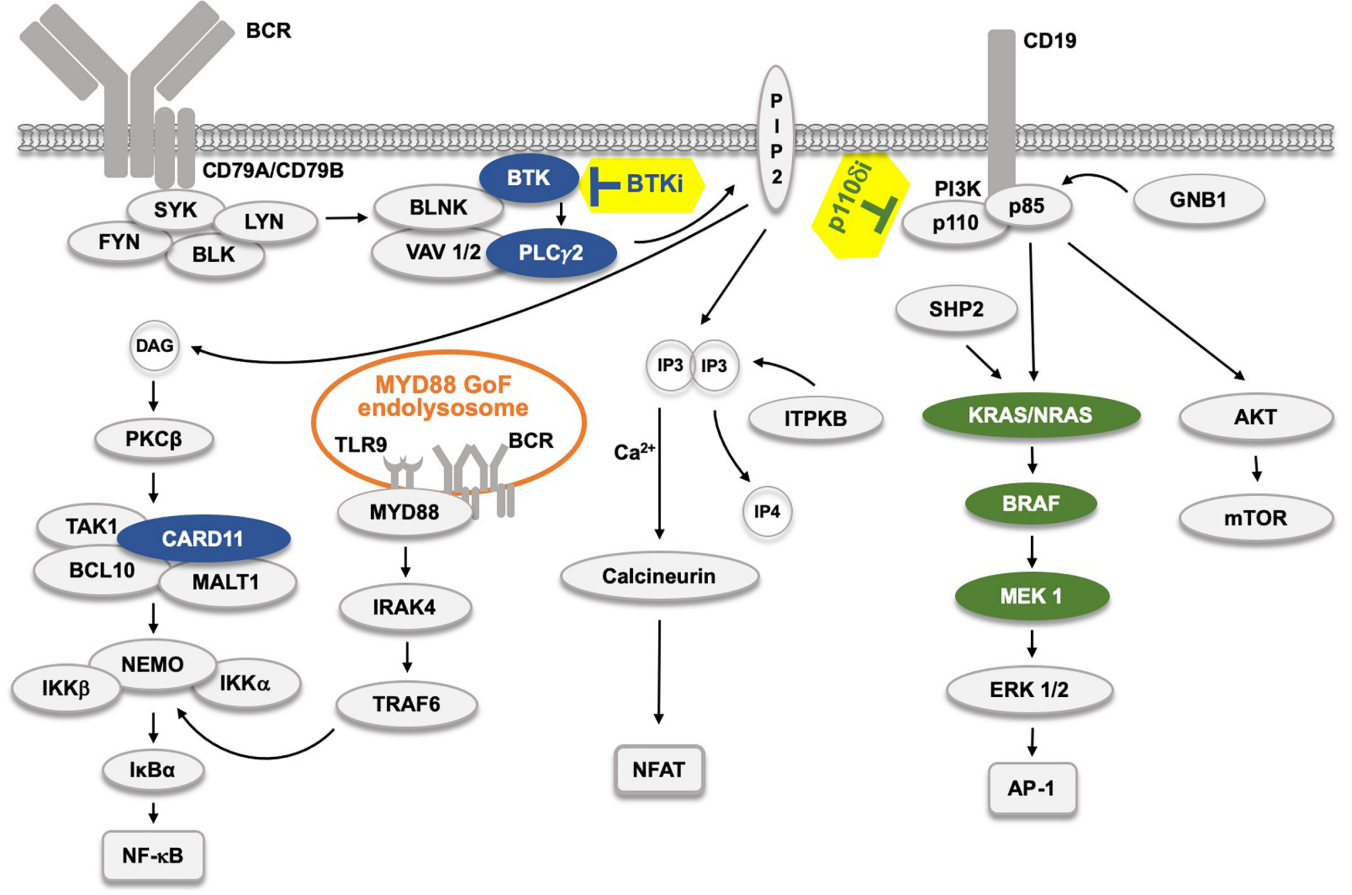
The B cell receptor (BCR) signaling pathway and the effect of kinase inhibitors. The BCR pathway trifurcates into, from left, NF-κB, NFAT and AP-1 signaling. The AP-1 pathway is believed to be connected to the BCR through LYN, SYK and ZAP70 (not depicted). Filled protein symbols correspond to Gain-of-Function (GoF) drug resistance mutations. BTK inhibitors (BTKi) and p110δ inhibitors (p110δi) are marked with yellow background. The MYD88 L265P GoF mutation found in 95% of WM patients, and also in ABC-type DLBCL, and in some CLL patients induces an activated endolysosome containing TLR9 and the BCR. 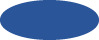 proteins affected by BTKi, 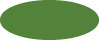 proteins affected by p110δi.

**Figure 2:**
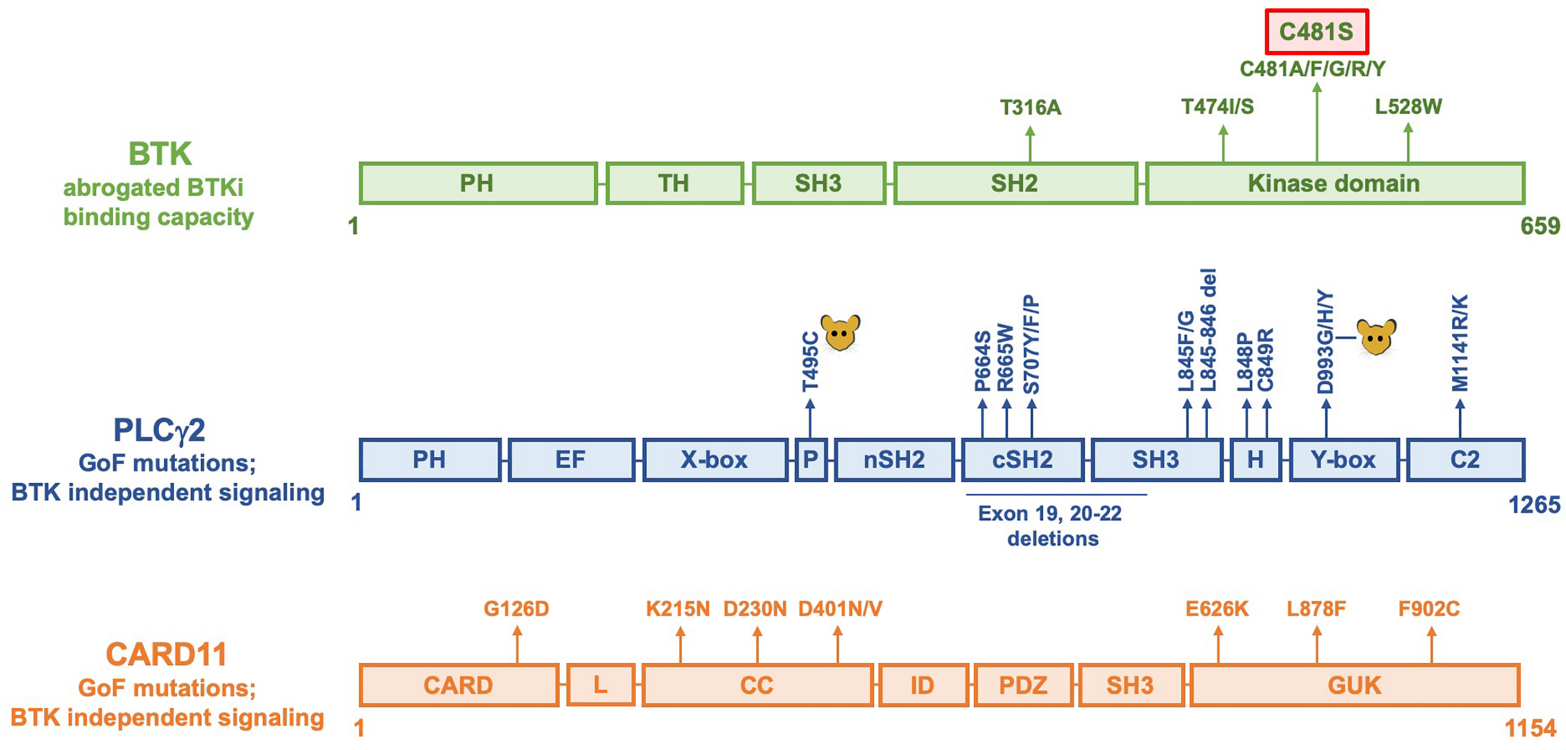
Linear representation of BTK, PLCγ2 and CARD11 molecules showing amino acid replacements causing resistance to BTKi. The C481S replacement is the most common alteration and is marked with a red box. In PLCγ2, alterations induced by mutagenesis in mice are indicated as 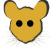. Not all reported alterations are listed, only recurrent mutations have been included.

In 2014, the first BTKi resistance mutations were reported. They were identified in CLL patients receiving ibrutinib treatment (51, 52). They fell into two categories: [1] point mutations that disable covalent binding of ibrutinib to BTK, by replacing the ibrutinib-target, cysteine 481 (C481) in the kinase domain (**Figures 1** and **2**), with a different amino acid, and [2] mutations causing constitutive activation of PLCγ2, the downstream substrate of BTK (variations altering PLCγ2 are described in detail in the next section). BTK mutations mainly originate from single base changes yielding substitution not only to serine, but also to arginine, phenylalanine, tryptophan or tyrosine (53). Predictions suggested that glycine substitutions also may occur (54), and indeed, later, glycine substitution of C481 was found in patients (55).

The C481S, and also the experimentally generated, C481T, threonine substitution were shown to maintain kinase activity (51, 52, 54), whereas the other substitutions are only weakly active (glycine) or kinase-dead (arginine, phenylalanine, tryptophan, tyrosine) (54). To this end, knock-in mice carrying serine 481 in their germline have a phenotype essentially identical to wildtype animals (56). Thus, this demonstrates a normal function of BTK harboring a serine at the 481 position.

The observation that *in vivo*-selection of *kinase-dead* mutants occurs, may be unexpected; the underlying mechanism has been discussed elsewhere (54). The C481 mutants are not exclusively seen during ibrutinib treatment, they are also found in patients treated with other irreversible BTKi that target C481, namely acalabrutinib (57) and zanubrutinib (58). Furthermore, resistance mutations not only appear in CLL, but also in MCL (59), marginal zone leukemia (MZL) (60) and Waldenström’s macroglobulinemia (14, 61).

A second site with amino acid substitutions affects the gatekeeper residue, threonine 474 (**Figure 2**). These replacements are more rarely seen during BTKi therapy, with only two exchanges reported to date, namely to isoleucine or to serine (53). However, the gatekeeper residue is the predominantly substituted amino acid in patients developing resistance against most other kinase-inhibitors, such as those directed against ABL in chronic myeloid leukemia and against EGFR in lung cancer [reviewed in (62)].

In a recent study, mutations affecting T474 and those yielding the C481S replacement were combined (18). Surprisingly, certain double mutants demonstrated super-resistance tolerating more than 16-fold the therapeutic BTKi concentration. Even more unexpected was the fact that C481S combined with T474A restored sensitivity to ibrutinib.

As depicted in **Figure 2**, rare recurrent replacements have also been reported affecting the leucine 528 in the catalytic domain of BTK (53, 58, 63) as well as threonine 316 in the SH2 domain (64, 65).

Furthermore, apart from the BCR-signaling components which are affected, there are additional resistance mutations. Those located to the short arm of chromosome 8 have been suggested to impair apoptosis by causing haploinsufficiency for the TRAIL receptor (61, 66), whereas other mutations affect additional genes (61, 67). *Del*(8p) was found in a sizable number of ibrutinib-resistant CLL cases, resulting in deletion and haploinsufficiency of *tumor necrosis factor-related apoptosis-inducing ligand-receptor* (TRAIL-R) (66, 68). This was associated with a decrease in the TRAIL-R mRNA and protein levels which corresponded to the size of the *del*(8p)-harboring clone. Interestingly, a potential indirect mechanism that would link TRAIL resistance to positive selection by ibrutinib therapy is suggested by the fact that TRAIL concentrations are higher in circulating blood as compared with the lymph node environment. Ibrutinib therapy is known to mobilize CLL cells from the lymph nodes into to the peripheral blood. Hence, haploinsufficiency of TRAIL-R could provide a survival advantage for CLL cells by making such CLL cells less sensitivity to cell death in the periphery, once released from the lymph node and exposed to higher levels of TRAIL.

BTK’s best known role in the BCR signaling pathway is to phosphorylate tyrosine residues in PLCγ2 (69, 70). GoF mutations affecting PLCγ2 therefore obviate the need for posttranscriptional phosphorylation, and, hence, BTK becomes redundant when malignant B cells acquire this mode of resistance to BTKi.

## PLCγ2 Resistance Mutations for BTKi

Two different amino acid substitutions, which caused PLCγ2 BTKi resistance mutations, arginine 665 to tryptophan and leucine 845 to phenylalanine, were presented in the first report on BTKi resistance (51). They were classified as GoF (51, 71), and, in line with this, activating germline mutations in the *PLCG2* gene located in chromosome 16q23.3, with dominant inheritance, were published already two years earlier (72). Not all *PLCG2* mutations found causing BTKi resistance have yet been reported to also induce PLCγ2-associated antibody deficiency *with* immune dysregulation (APLAID), or *without* autoinflammation (PLAID), but many do. The most recurrent mutations are depicted in **Figure 2**. Even prior to the identification of constitutively active PLCγ2 in humans in 2012, GoF mutations affecting PLCγ2 were observed in mice following N-ethyl-N-nitrosourea mutagenesis (73). They occur in the same domains, which are affected by both BTKi resistance and germline *PLCG2* mutations in humans. The first reported alteration in mice caused an aspartate 993 to glycine replacement in the C-terminal part of the catalytic domain, known as the Y-box (73). Later a mutation was identified in the split pleckstrin homology (PH) domain yielding a tyrosine 495 to cysteine substitution (74). Both mutant strains develop severe spontaneous inflammation and autoimmunity (73–75).

PLCγ2 is a large molecule with a M.W. of 147,870 Da, i.e. more than twice the size of BTK. It consists of 10 domains as depicted in **Figure 2**. The activation state of the mutated PLCγ2 has been suggested to be a form of pre-activation, rather than constitutive activation (76, 77). However, constitutive activation does not necessarily mean full activation, and, hypothetically, if the constitutive activation is too strong, the affected cell, or its host, may not survive long-term.

## CARD11 (CARMA1) Resistance Mutations for BTKi

CARD11, also known as *caspase recruitment domain-containing membrane-associated guanylate kinase protein*-*1* (CARMA1), is an adaptor protein, which forms an integral part of BCR signaling (**Figures 1** and **2**). CARD11 is autoinhibited in its natural state, but upon activation the latch domain is released, and the protein forms a complex with *B-cell lymphoma/leukemia 10* (BCL-10) and *mucosa-associated lymphoid tissue lymphoma translocation protein 1* (MALT1), generating the CBM scaffold, which can oligomerize (78). The activation is initiated by PKCβ, which phosphorylates a series of serine sites in the CARD11 inhibitory domain, the initial modification necessary for the assembly of the CBM complex (79, 80). The serine phosphorylation alters CARD 11 into an open conformation accessible for BCL10-MALT1 binding.

CARD11 mutations in tumors were first reported in DLBCL in 2008 and transfer of the identified CARD11 coiled-coil domain mutants into lymphoma cell lines resulted in constitutive NF-κB activation (81). As depicted in **Figure 2**, the leucine 878 to phenylalanine resistance mutation to BTKi has been found in CLL, MCL as well as in WM and several more in MCL (82, 83). CARD11 is located downstream of BTK in the BCR pathway, but upstream of NF-κB, whose signaling is crucial for B-cell survival. In a set of follicular lymphomas treated with ibrutinib, CARD11 mutations were significantly associated with resistance (84).

Inherited mutations in CARD11 are associated with different diseases. Thus, heterozygous dominant-negative variants of CARD11 result in combined immunodeficiency with, or without atopy, high IgE levels and other autoimmune features [reviewed in (85, 86)]. Germline GoF CARD11 mutations instead cause *B cell expansion with NF-κB and T cell anergy* (BENTA), accompanied by elevated NF-κB activity and hyperproliferative B cells (87). Very recently, more than two thousand CARD11 variants were generated by multiplexed, CRISPR-mediated, saturation genome editing, functionally scored and classified (88). A single LoF mutation affecting BCL-10 has been identified, whereas none has been reported in MALT1, and GoF variants were not published for any of them (89).

Collectively, this clearly demonstrates that CARD11 is a complex molecule with a multitude of activities. Importantly, together with PLCγ2 it is the only protein in which GoF mutations have been repeatedly found to cause resistance to BTKi.

## Lack of BTKi Resistance Mutations in the PI3K-RAS and Calcineurin Arms of the BCR Pathway

The B cell malignancies that respond well to BTKi, especially CLL, are particularly dependent on BCR pathway activation for growth and survival (24). This goes beyond tonic BCR signaling that all B cells need for survival, and is induced, presumably by activating conformational BCR properties or tissue auto-antigens in the supporting microenvironment of secondary lymphatic organs (i.e. spleen and lymph nodes). Activating mutations upstream of BTK will be inhibited by BTKi, whereas downstream GoF alterations would be insensitive. Based on this, it could be hypothesized that any component downstream of BTK in this signaling pathway could induce unresponsiveness to BTKi when constitutively active. Of note, the signaling downstream of PLCγ2 diverges into diacylglycerol (DAG)- and inositol 1,4,5-trisphosphate (IP3)-dependent pathways and it is therefore conceivable that GoF mutations would need to affect both of these arms. However, this is not the case in B-cell malignancies, since to date resistance mutations have only been reported in the DAG pathway (**Figure 1**).

Hence, after the initial phase, in which PLCγ2 generates diacyl glycerol (DAG) and inositol 3-phosphate (IP3), BCR signaling bifurcates into the DAG and IP3 pathways, and subsequently trifurcates since DAG activates RAS (not depicted) and NF-κB (**Figure 1**). The BCR connection to RAS is likely mainly through CD19 and PI3K. Hence, LYN, SYK and ZAP-70 have been shown to phosphorylate the YXXM motif in CD19, to which PI3K can tether (90–92). Moreover, generation of phosphatidylinositol (3–5)-trisphosphate (PIP3) by PI3K is essential for the activity of and membrane tethering of BTK (93–95). While resistance mechanisms affecting the PI3K-AKT-FOXO3a axis, without involving genetic alterations, may occur (96–98), including transcriptional reprogramming in MCL (99), it is still highly unexpected that GoF substitutions affecting the corresponding signal transducers would be absent.

Interestingly, GoF resistance mutations to BTKi are confined to the protein kinase Cβ (PKCβ) arm, although variations in PKCβ itself do not seem to cause resistance. Concerning the RAS-ERK pathway, in which BTKi resistance mutations were not reported, *primary* mutations causing RAS GoF are the hallmark of numerous forms of neoplasms (100). They have been found in CLL but seem unrelated to BTKi treatment (64). Furthermore, nor have any resistance variants been accounted for in the calcineurin arm (**Figure 1**). It should be mentioned that a cysteine to phenylalanine substitution at amino acid 873 in inositol-trisphosphate 3-kinase B (ITPKB) has been reported under ibrutinib-treatment (68). However, this alteration was identified in a patient with Richter’s transformation and variations are also known in DLCBL. Lack of ITPKB is manifested at the transition from immature to transitional B-cells (101). Thus, while this mutation potentially could affect the calcineurin arm, there is no clear evidence that it is related to BTKi resistance.

This does not in any way mean that the RAS and calcineurin pathways are unimportant for B-cells; it could merely reflect that they are redundant in the leukemias and lymphomas, which are sensitive to treatment with BTKi. In conclusion, acquisition of GoF mutations seems to be rare among BTK downstream signaling components. As described in the next section, the lack of such GoF mutations in transformed B-cells is even more surprising, since they do occur in malignant T-cells.

## PKCβ and VAV1 GoF Variants Are Drivers in Adult T Cell Malignancy

**Figure 3:**
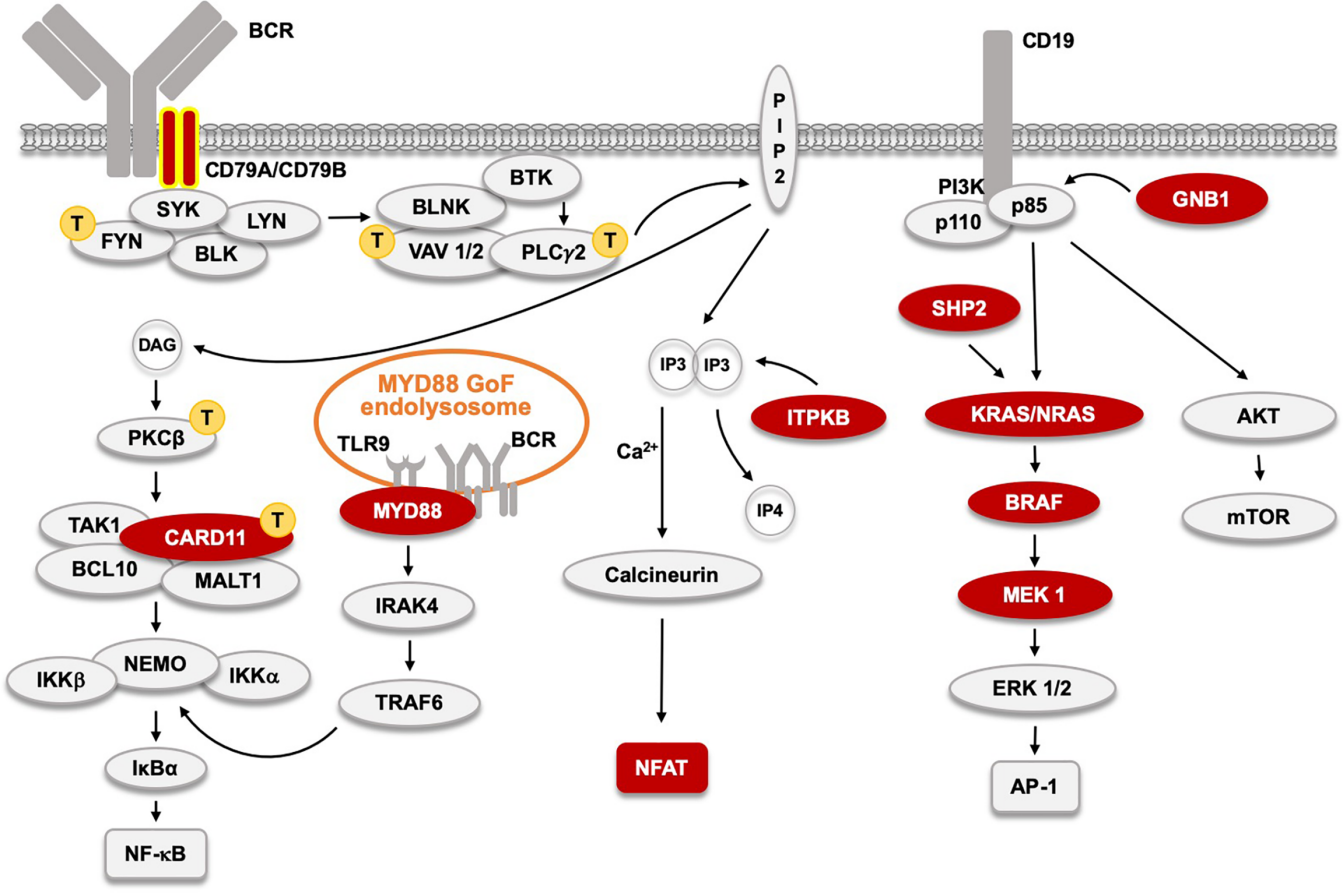
The B cell receptor (BCR) signaling pathway and acquired, primary, driver Gain-of-Function (GoF) mutations found in CLL and other BTKi-sensitive malignancies 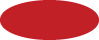. CD79A and B GoF, found in WM and ABC-type DLBCL, and very rare in CLL, are labeled with yellow contour. Signaling proteins marked with 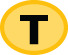 indicate those affected by primary GoF mutations in adult T cell leukemia as reported (103). The MYD88 L265P GoF mutation in 95% of WM patients, and also in ABC-type DLBCL, and in some CLL patients induces an activated endolysosome containing TLR9 and the BCR.

Adult T cell leukemia/lymphoma (ATL) is a highly malignant, retrovirus-induced cancer caused by HTLV-1. It is endemic in certain parts of Japan, the Caribbean and Africa (102). In a study of 426 ATL patients, activating mutations were found in a large proportion (103). These variants are depicted in yellow with a T in **Figure 3**. Similar to what has been observed for BTKi unresponsiveness in B cell malignancies, GoF mutations affecting PLCγ were also identified in ATL. These alterations were found in PLCγ1, which is the isoform expressed in T-cells, whereas in B-cells the corresponding form is PLCγ2. Furthermore, acquired GoF variants were observed in CARD11, as also noted in some patients treated with BTKi.

However, a number of the GoF mutations found in ATLs affected proteins for which variants were never reported to cause resistance to BTKi, namely PKCβ, FYN and VAV1 (**Figure 3**). Regarding FYN, activating SRC-family kinases are located upstream of BTK, and hence alterations in FYN, LYN or BLK would not be expected to cause resistance to BTKi.

ATL is the first tumor in which recurrent GoF alterations in the *PRKCB* gene have been described (103). This was unexpected, in particular for the reason that PKCθ is known to the most important isoform in T-cell receptor signaling (104). More than 90% of the mutations affecting PKCβ in ATL were confined to a conserved region of the catalytic domain. In contrast, PKC *LoF* mutations have been identified in many solid tumors. The PKC family contains 9 genes, and in a comprehensive study, 46/554 of the identified PKC mutations were selected for functional analysis (105). The absolute majority was found to cause LoF and none was activating.

**Figure 4:**
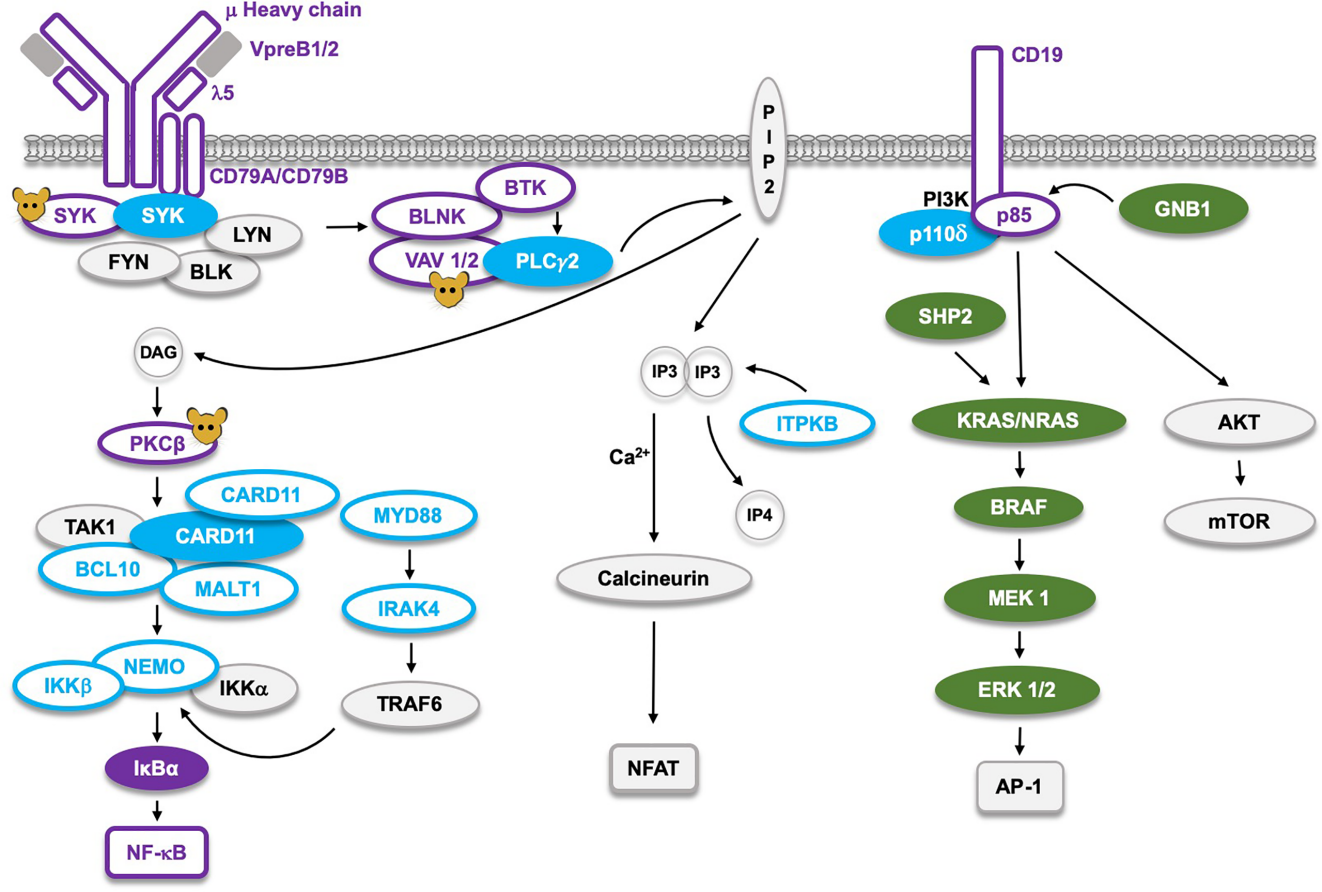
The B cell receptor (BCR) signaling pathway and the corresponding germline mutations found in man, or only generated in mice (molecules marked with 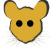). Proteins marked as 
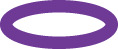
 represent Loss-of-Function (LoF) mutations with defects *limited to B cells*, whereas the LoF mutations marked as 
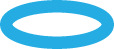
 cause defects *not* limited to B cells. The corresponding filled symbols mark Gain-of-Function (GoF). CARD11 and SYK, which are the only proteins affected by either GoF or LoF, appear as two molecules. LoF mutations in the NF-κB pathway cause B cell defects, whereas those affecting AP-1 signaling result in GoF RASopathies marked as 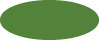. The single LoF mutation in the NFAT pathway affects the *ITPKB* gene causing severe combined immunodeficiency (SCID), primarily influencing development of the T cell lineage.

While heritable LoF or GoF mutations affecting PKCβ have not been reported in humans (**Figure 4**), inactivation of the corresponding gene in the mouse germline causes a phenotype similar to BTK deficiency (106). As mentioned, GoF mutations affecting PKCβ frequently occur in ATL. It is therefore likely that spontaneous GoF mutations in the *PRKCB* gene, would have occurred multiple times in B-CLL, but seemingly without providing any survival advantage for the affected cell. Identifying the reason for this conundrum would be of great interest to further our understanding of BCR-signaling, including in CLL.

The VAV guanine nucleotide exchange factor is, similar to PKCβ, positioned downstream of BTK, and could therefore potentially cause resistance to BTKi. However, alterations in this gene family have so far not been reported, suggesting that, if they at all exist, they must be very rare. In contrast, VAV1 GoF mutations clustering into hotspots in the acidic, zinc-finger and SH3 domains were found in ATL (103), which demonstrates that they do occur and are selected for. They disable an autoinhibitory mechanism and are considered to cause constitutive activation. Mice with inactivation of the genes encoding VAV1- 2- and -3 in the germline (**Figure 4**) fail to produce significant numbers of recirculating follicular or marginal zone B cells (107). Similar to the situation for PKCβ, it would be interesting to understand why there is an absence of detectable VAV1 GoF variants in CLL, since they do appear in ATL.

## The Origin of the Resistance Mutation Affects Leukemic Clone Size and Variant Allele Frequencies

The analysis of the size of resistance clones has demonstrated interesting differences. Although there is considerable heterogeneity among patients, mutations affecting BTK itself show higher allele frequencies than those affecting PLCγ2 or CARD11, and the resulting clone size is often also larger. It is common that several resistance variants exist in the same patient, such as C481S combined with C481Y, or C481S combined with a GoF mutation in the *PLCG2* gene, for example. It has also been found that BTK C481S mutants can occur in the same patient and result from different changes in the TGC codon for cysteine, to AGC or to TCC, both of which encode serine (108). While in this case it was possible to prove different origins because of sequence differences, the identical mutation in which the TGC cysteine codon is altered to the same codon for serine, could also occur in different CLL clones as recurring events. Collectively this demonstrates that resistant clones in the same patient can arise repeatedly during the evolution of the malignant B cell clone, and that the frequency by which they occur likely is underestimated. Since resistance mutations are random events, the highest number of mutational events is expected to exist at the time when the tumor burden is the largest (62), which often is prior to therapy.

To this end, careful dissection of the evolution of CLL under the treatment with BTKi established that resistance mutants were present already prior to therapy (66). This was predicted based on calculation of the leukemia cell burden and other parameters at the onset of treatment (109). During ibrutinib therapy it was estimated that 1.7% of *blood* CLL cells and 2.7% of *tissue* CLL cells die per day, 3-5 times higher rate than without treatment (110). This suggests that the ibrutinib-induced reduced tissue disease burden is primarily caused by CLL cell death and to a lesser degree due to CLL cell egress from nodal compartments.

These findings do, however, not explain the reason for that resistance mutations affecting BTK are more frequent and yield a larger clone size than those affecting PLCγ2 or CARD11. Owing to that mutations affecting PLCγ2 and CARD11 are known to occur in multiple sites along the molecules, the mere lack of residues suitable for replacement cannot be the underlying mechanism. Current knowledge (**Figure 1**) suggests a crucial role for PLCγ2 in the generation of diacylglycerol (DAG) and inositol 1,4,5-trisphosphate (IP3), which subsequently activate PKCβ, and intracellular calcium-signaling, respectively. However, it is possible that it is not sufficient to only activate PLCγ2, but that BTK has yet another role beyond DAG- and IP3-mediated signaling.

## Recurrent *Primary* Mutations in B Cell Malignancies Responding to BTKi

While the focus of this review is on resistance mutations to BTKi, it is of interest to compare these alterations with those found as recurrent *primary* mutations. Genetic alterations are the basis for tumorigenesis, including leukemogenesis. How neoplasms evolve with regard to their mutation spectrum over the course of disease and therapy is key to the understanding of cancer biology. Interestingly, the primary mutations differ substantially among BTKi sensitive tumors.

Hence, in CLL the majority of the *primary* driver mutations are unrelated to BCR signaling proteins with alterations affecting the *MIR15A* and *MIR16-1*, *ATM*, *BIRC3*, *NOTCH*, *SF3B1*, and *TP53* genes being highly prevalent (111–113). In contrast, in WM, the genetic changes mainly affect the BCR pathway, with 95% of patients carrying the MYD88 leucine 265 to proline substitution, L265P (114, 115). Mutations affecting the G-protein coupled receptor CXCR4 are also prevalent in WM, but not in CLL (115). However, MYD88 mutations are overrepresented in CLL (112, 113) and they are more often clonal than subclonal (116).

Even if MYD88 is not part of the classical BCR pathway it activates canonical NF-κB signaling (117). MYD88 is an adaptor protein mediating signals from Toll-like and interleukin 1 receptors. It forms a supramolecular complex, known as the Myddosome (118). As depicted in **Figures 1** and **3**, in both ABC-type DLBCL and WM, a MYD88-TLR9-BCR (My-T-BCR) complex is generated at the endolysosome, while this is not characteristic of MCL (116). My-T-BCR connects MYD88 to IgM, PLCγ2, the CARD11-BCL10-MALT1 complex and to mTOR, and the L265P substitution in MYD88 is crucial for generating such a constitutively active signaling hub. In animal experimental model systems TLR9 acts together with BTK to induce autoimmunity (119). Remarkably, *primary* GoF mutations affecting components downstream of MYD88, such as IRAK4 have not been described in WM.

In WM, gain-of-function mutations instead affect CD79B (Igβ), which is an essential upstream signaling module of the BCR complex (115),. In the ABC subtype of DLBCL GoF alterations mainly also affect CD79B, but they are found in CD79A, too (116). In CLL, GoF mutations in CD79A and B exist, but are very rare (112, 113).

In CLL, *primary* GoF, driver mutations are found in components of not only the NF-κB but also of the RAS-MAPK pathway, as depicted in **Figure 3** (112, 113, 120). The very recent report demonstrating that resistance mutations to inhibitors of p110δ selectively affects the MAPK pathway in CLL patients is highly interesting (121). It shows that also this arm of the BCR pathway can be affected by resistance mutations (**Figure 1**), although they never appear after BTKi treatment. The existence of such resistance mutations in CLL upon p110δi therapy suggests that the PI3K arm of BCR signaling primarily affects RAS-MAPK signaling. Furthermore, these findings suggest that combined treatment with BTKi and p110δi may give synergistic effects, and such studies have already been performed (122, 123).

Finally, the third arm of BCR signaling is also affected in CLL. This pathway is formed by PLCγ2-generated *inositol 1,4,5-trisphosphate* (IP3) activating the phosphatase calcineurin, which subsequently de-phosphorylates the transcriptional regulator *nuclear factor of activated T-cells*, NFAT (**Figures 1** and **3**). De-phosphorylation of NFAT results in nuclear translocation and enhanced nuclear presence of NFAT is observed in CLL (124). The underlying mechanism involves hypomethylation of the *NFATC1* promoter (125), and the degree of NFAT activation has been reported to predict the clinical outcome in CLL (126).

Proteins involved in the adhesion and migration of leukemia and lymphoma cells have demonstrated their importance in both CLL and MCL (127, 128). However, with the exception of CXCR4 in WM (115), the corresponding *activating mutations* have not been identified. Notably, in both CLL and MCL adhesion molecules, including CXCR4, are affected by BTKi treatment (127, 129, 130). In WM somatic *CXCR4* mutations were reported to activate AKT and ERK (131).

## Phenotype of Germline, Loss-of-Function Mutations Affecting Components of the BCR Pathway

Mutations in leukemia and lymphoma, which cause resistance to treatment with kinase inhibitors, mainly are GoF. Importantly, only a subset of these proteins is also frequently affected by *primary* GoF mutations in B cell malignancies (**Figures 1** and **3**). For NF-κB signaling these are CD79A, CD79B, CARD11 and in particular MYD88. A likely explanation for this selectivity is that many of the molecules in this pathway simply are not susceptible to GoF activity through a mutational event. This seems to be the case for e. g. BTK and the adaptor BLNK for which constitutively activating variants are lacking. Conversely, as depicted in **Figure 4**, the corresponding LoF mutations cause very severe B cell deficiency (1, 132, 133).

Recently (**Figure 4**), inherited GoF mutations were observed in the *SYK* gene (134). They cause a B cell defect combined with a multiorgan, inflammatory disorder and patients do not develop CLL, but, instead, an increased frequency of DLBCL. While having driver activity, primary SYK mutations are very rare among DLBCL patients (135, 136), but are increased in many cancer cell lines (137). Moreover, in peripheral T cell lymphoma the BTK-related ITK kinase, upon a translocation event, forms a fusion protein with SYK (138), which induces STAT3 phosphorylation and CD69 expression (139).

In **Figure 4**, components of the BCR causing hereditary disease are highlighted. In the NF-κB pathway LoF mutations predominantly induce defects in B cell development. This is true for all the components of the pre-BCR and several of the downstream signaling proteins. BTK is highly conserved during evolution (140) and mutations in the *BTK* gene are the most frequent, since this gene is located on the X-chromosome making boys particularly susceptible (1, 133, 141). Single LoF mutations affecting the SRC-family kinases, BLK, FYN and LYN do not cause B cell deficiency, while a combined triple-knockout in mice does (142). Whereas LoF alterations for PLCγ2 have not been described in humans, activating mutations cause inherited hyperinflammation as depicted in **Figure 2**.

LoF mutations affecting VAV or PKCβ proteins have not been identified in the germline of humans, likely because more than a single gene needs to be inactivated. SYK LoF is lethal in mice but has not been observed in humans. In addition to an endothelial abnormality, it causes a severe B-lineage defect in mice (143, 144). Thus, it belongs to the group of proteins whose genes have been inactivated in mice, but for which LoF variants were never reported in humans (marked with a mouse symbol in **Figure 4**).

Mutations in components of the CARD11-BCL10-MALT1 complex affect the development of the B cell lineage but also give rise to other phenotypes (86, 89). CARD11 is so far unique in that both GoF and LoF germline mutations have been identified *in humans*, with LoF causing a combined immunodeficiency affecting both B- and T-cells (86, 89, 145). The inhibitory IκBα can be affected with GoF variations, whereas NF-κB only shows LoF and these cause B cell deficiency (145). LoF affecting MYD88 mainly affects innate immunity and patients surviving bacterial infections early in life develop adaptive immunity protecting them from subsequent infectious episodes (145, 146).

Inherited variations in the calcineurin-NFAT pathway have not been reported. However, LoF mutations in the *inositol-trisphosphate 3-kinase B* gene, *ITPKB*, cause severe combined immunodeficiency (**Figure 4**). While B cells secondarily are influenced by this loss, the defect primarily affects T cells (147). Moreover, GoF alterations in this gene belong to the group of primary mutations found in CLL (**Figure 3**).

Germline mutations in the RAS-MAPK pathway (**Figure 4**) cause RASopathies, many of which belong to the Noonan syndrome (148). RASopathies are characterized by unusual facial features, congenital heart disease, growth and skeletal abnormalities, intellectual disability and predisposition to neoplastic development. Of note, the overlap between primary mutations in CLL (**Figure 3**) and those providing resistance (**Figure 1**) is so far only partial. Thus, activating mutations affecting SHP2 and GNB1 exist as primary alterations in CLL and cause RASopathy, but have not as yet been found to induce resistance to p110δi. Inherited GoF mutations affecting p110δi cause a combined immunodeficiency and hyperinflammatory state (149), whereas LoF defects of its partner p85 result in a primary B cell defect (145).

Collectively this demonstrates that there is an interesting, but complex, interplay between signaling proteins, with often major phenotypic differences between germline and acquired primary mutations in B-lineage cells. Moreover, the patterns that are being revealed through mutational analysis of both primary and drug-induced resistance provide important insights into leukemogenesis.

Of further note are the RAS-MAPK pathway mutations found in CLL, since they clearly exemplify that the phenotype of germline mutations and those found in tumors do not necessarily overlap. It can be anticipated that every single gene likely is mutated as a rare event in every tumor type (but not in every patient). Because cells with an acquired *driver* mutation will be selected for, this experiment of nature, suggests that continued sequencing of tumor genomes eventually will reveal every key component in tumor biology. Because of the dependence on additional genomic alterations for the selection of cells having acquired a potential driver mutation, revealing the interplay is a formidable task. Drug treatment and the selection of resistance mutations will add another layer of information, which may aid in this process.

## Concluding Remarks

The common linear representation of the BCR-signaling pathway, which trifurcates into the NF-κB, NFAT and AP-1 arms, does not fully explain the observed pattern of resistance mutations in patients treated with BTKi. Although GoF mutations affecting both PLCγ2 and CARD11 are compatible with such a scheme, the lack of PKCβ and VAV GoF variants is not. Furthermore, resistance mutations in the NFAT or the AP-1 arms have not been observed in patients treated with BTKi.

Similarly, in WM, why is there such a predominance of *primary* driver mutations generating an activated form of MYD88? Why are there no examples of *primary* GoF driver mutations affecting proteins downstream of MYD88, such as IRAK4? This indicates that MYD88 has some unique properties apart from activating IRAK1, IRAK4 and TRAF6 mediated signaling. Alternatively, these components may not be susceptible to GoF.

Inherited SYK GoF mutations frequently cause DLBCL, whereas *inherited* GoF mutations of CD79A and B were not yet reported. In contrast, CD79A and B *primary* mutations in tumors are found in CLL, WM as well as in DLBCL, whereas primary SYK mutations are very rare, calling into question a strict linear signaling relationship.

A conundrum has been the mechanism underlying the variant allele frequencies among resistant clones. Thus, replacement of cysteine 481 is the most frequent alteration and the corresponding clones are usually also the largest. This observation suggests a gradual reduction of the biological activity of individual proteins reflecting their position in the descending BCR pathway. The recent finding that the arms of the BCR pathway are differentially affected by BTKi and p110δi, is compatible with this idea and also has therapeutic implications in favor of combination therapies.

Hence, although the NF-κB, NFAT and AP-1 downstream signaling arms from the BCR appear in the form of textbook information, the resistance patterns observed in patients treated with BTKi and p110δi reveal that there is more to learn. For example, precisely how the BCR is connected to PI3K signaling needs further investigation as well as the role of PI3K-regulated AKT and mTOR. This is key for the basic understanding of the BCR multicomponent signalosome, and also for providing optimal therapeutic solutions, since combination therapies may prevent resistance from developing.

## Author Contributions

CS determined the scope and focus of the review and drafted the manuscript and generated the figures. JB wrote part of the manuscript and provided critical feedback. Both authors contributed to the article and approved the submitted version.

## Funding

This work was supported by the Swedish Cancer Society, Stockholm County Council (ALF-project) and Swedish Medical Research Council. This work was supported in part by the generous philanthropic contributions to The University of Texas MD Anderson Cancer Center Moon Shots Program™, the CLL Global Research Foundation, and the MD Anderson Cancer Support Grant CA016672.

## Conflict of Interest

JB reports honoraria from consulting/an advisory role with Janssen; research funding from Gilead, Astra Zeneca, BeiGene, and Pharmacyclics LLC, an AbbVie Company; travel, accommodations, or other expenses from Gilead and Janssen.

CS declares that the research was conducted in the absence of any commercial or financial relationships that could be construed as a potential conflict of interest.
